# Detection of radiosensitive subpopulations *ex-vivo* with Raman microspectroscopy

**DOI:** 10.3389/fonc.2025.1470431

**Published:** 2025-02-27

**Authors:** Aidan D. Meade, Adrian Maguire, Jane Bryant, Daniel Cullen, Dinesh Medipally, Lisa White, John Armstrong, Mary Dunne, Emma Noone, Shirley Bradshaw, Marie Finn, Aoife M. Shannon, Orla L. Howe, Fiona M. Lyng

**Affiliations:** ^1^ School of Physics, Clinical and Optometric Sciences, Technological University Dublin, Dublin, Ireland; ^2^ Radiation and Environmental Science Centre (RESC), Physical to Life Sciences Research Hub, FOCAS Building, Technological University Dublin, Dublin, Ireland; ^3^ School of Biological, Health and Sport Sciences, Technological University (TU) Dublin, Dublin, Ireland; ^4^ Department of Radiation Oncology, Saint Luke’s Radiation Oncology Network (SLRON), St Luke’s Hospital, Dublin, Ireland; ^5^ Cancer Trials Ireland, Dublin, Ireland

**Keywords:** vibrational spectroscopy, radiosensitivity, ataxia telangiectasia, non-Hogkin’s lymphoma, Turner’s syndrome, principal components analysis, universal manifold approximation and projection, support vector machine

## Abstract

Although significant advances in understanding the molecular drivers of acquired and inherited radiosensitivity have occurred in recent decades, a single analytical method which can detect and classify radiosensitivity remains elusive. Raman microspectroscopy has demonstrated capabilities in the objective classification of various diseases, and more recently in the detection and modelling of radiobiological effect. In this study, Raman spectroscopy is presented as a potential tool for the detection of radiosensitivity subpopulations represented by four lymphoblastoid cell lines derived from individuals with ataxia telangiectasia (2 lines), non-Hodgkins lymphoma, and Turner’s syndrome. These are classified with respect to a population with mixed radiosensitivity, represented by lymphocytes drawn from both healthy controls, and prostate cancer patients. Raman spectroscopic measurements were made *ex-vivo* after exposure to X-ray doses of 0 Gy, 50 mGy and 500 mGy, in parallel to radiation-induced G2 chromosomal radiosensitivity scores, for all samples. Support vector machine models developed on the basis of the spectral data were capable of discrimination of radiosensitive populations before and after irradiation, with superior discrimination when spectra were subjected to a non-linear dimensionality reduction (UMAP) as opposed to a linear (PCA) approach. Models developed on spectral data acquired on samples irradiated *in-vitro* with a dose of 0Gy were found to provide the highest level of performance in discriminating between classes, with performances of F1 = 0.92 ± 0.06 achieved on a held-out test set. Overall, this study suggests that Raman spectroscopy may have potential as a tool for the detection of intrinsic radiosensitivity using liquid biopsies.

## 1 Introduction

Cancer incidence is projected to increase globally by 47%, from 18.1 million to 29.5 million annually, over the period from 2020 to 2040 (1). Consequently an associated increase in the numbers of patients requiring radiotherapeutic treatment is expected, if the proportion of patients receiving radiotherapy as part of their care remains constant at ~50% (2). While documented increases in incidence of cancer partially stems from development of technologies for detection and diagnosis of malignancies, the increase in incidence has driven the need for more efficient and effective treatment methodologies. In recent years many efforts have been made in the advancement of personalized treatment in radiotherapy and while some success in this area has been made, mainly due to the advancement of technologies associated with confining dose delivery to malignant regions, less success has been made in advancement in biological assays to assess for inter-individual variation in the response to radiotherapeutic treatment.

The dependence of the individual’s response to ionising radiation (IR) exposure on various innate genetic, epigenetic and other characteristics has yet to be fully characterised. Efforts have been made to study individual cases of extreme radiosensitivity in order to elucidate the cellular mechanisms involved in severe cases, in conditions such as Ataxia Telangiectasia, Nijmegen Breakage Syndrome and Fanconi’s Anemia, with therapeutic implications for patients undergoing radiotherapy treatment or for individuals subject to occupational and/or accidental irradiation (3). However, it has emerged that a distribution in intrinsic radiosensitivity exists in humans. Coles et al. demonstrated that the level of intrinsic radiosensitivity of the general population follows a Gaussian distribution, encompassing radioresistant, normal and radiosensitive individuals (4, 5). In their description we can understand extreme radiosensitivity in cases such as those associated with Ataxia Telangiectasia, as outlying cases within the distribution of intrinsic radiosensitivity. In addition, the existence of this phenomenon of inter-individual variation highlights the requirement for individual assessment of radiosensitivity to curtail dose delivery to patients undergoing radiotherapeutic treatment. Curtailing dose delivery would result in the reduction of normal tissue complications during radiotherapy, radiation-induced late effects of radiotherapy, and rule out radiotherapy as a treatment modality in cases of extreme radiosensitivity (6).

Ataxia Telangiectasia is a genetic neurodegenerative disorder resulting from a deficiency in the action or regulation of the gene Ataxia Telangiectasia Mutated (ATM), which is involved in the DNA damage response (DDR) pathway. Non-Hodgkin’s lymphomas (NHL) are radiosensitive tumours of the blood that have also been associated with deficiency in the repair of DNA damage through the ATM pathways (7, 8). In contrast Turner Syndrome is a cytogenetic condition in females whereby the X chromosome is partly or completely missing. Limited evidence exists that Turner syndrome is associated with increased radiosensitivity (9).

ATM is responsible for the sensing of DNA double strand breaks (DSBs) and plays an important role in pathways associated with the DDR and cell cycle checkpoint arrest (10, 11). ATM is one of the first proteins that localizes to the site of a DSB and phosphorylates the histone H2AX (γ-H2AX) and Ku proteins in response to detection of a DSB (12). These phosphorylated proteins then recruit further repair molecules to the site of the DSB. If unrepaired, DSBs may lead to cell cycle arrest, senescence or more severe outcomes such as genetic instability or programmed cell death (13, 14).

Since ATM phosphorylates H2AX in response to the formation of DSBs, the γ-H2AX assay has been widely used as an assay for the measurement of DSB sensing and repair (15, 16). While the assay is a good measure of both DNA damage and damage sensing of DSBs, its use in dosimetry and assessment of individual radiosensitivity has been hindered by the large variation in individual baseline levels of H2AX phosphorylation (17, 18).

An assay that has proven to be more reliable and reproducible in terms of dose response and as a measure of individual radiosensitivity is the *in-vitro* G2 chromosomal radiosensitivity assay (G2 assay) (19). The G2 assay measures, *in-vitro*, the number of chromosomal breaks (either spontaneously induced or induced as a result of exposure to IR) in cells in the G2 phase of the cell cycle before entering mitosis. Other metrics such as the Mitotic Index and Mitotic Inhibition can be calculated from this assay with or without exposure to IR. Mitotic Index is the ratio of the number of cells in metaphase versus the number of cells in interphase. Mitotic Inhibition is a measure of cell cycle checkpoint response to IR and is a measure of the increase or decrease in the number of cells in metaphase following IR when compared to the unirradiated control (20).

A wealth of recent and previous studies using both infrared and Raman spectroscopy have demonstrated that both measurements can identify and interpret radiobiological alterations in exposed cells, tissues and organisms (21–30). Raman spectroscopy uses the inelastic scattering of coherent light from a specimen, where the change in frequency of the light obtained is a fingerprint of the organic biochemistry of the system, and can be used to classify disease states (31), and biochemical processes associated with radiobiological response (32, 33).

In this study, a classification pipeline was developed using Raman spectroscopy of lymphocytes with machine learning approaches to identify radiosensitive cell populations. Here Raman spectra were acquired from lymphocytes drawn from a mixed population, including from the blood of healthy controls and prostate cancer patients, to represent a distribution of radiosensitivity. Spectra were also acquired from lymphoblastoid cell lines established from patients with a range of syndromes conferring innate radiosensitivity (Ataxia Telangiectasia (AT), Turner Syndrome and AT plus NHL (AT-NHL)). Although many spectroscopic studies on the effects of ionising radiation on various cell lines at various radiation doses (34), to the best of our knowledge this study represents the first which has utilized cellular models of radiosensitivity that are well characterised genetically, cytologically and radiobiologically.

Samples were irradiated with doses of 0 Gy, 50 mGy and 500 mGy to test whether innate spectral differences within the resting lymphocytes, or those emerging from irradiation, would provide discrimination of samples based on radiosensitivity. At these dose levels it is possible to decipher and differentiate on the molecular mechanisms associated with radiation sensitivity across cell and tissue types (35). In this study the radiation-induced G2 score radiosensitivity assay, which utilizes doses up to 500mGy, provided a cytological reference metric of radiosensitivity.

Machine learning classification models employing these spectra were developed using a support vector machine (SVM) algorithm to which spectral data was input after decomposition using principal component analysis (PCA) or universal manifold approximation and projection (UMAP). Our results indicate that incorporation of a UMAP decomposition step within the pipeline provides superior model performance to that achieved using PCA, suggesting a strategy for spectral pre-treatment within models for identification of radiosensitive individuals in practice.

## 2 Materials and methods

### Culture of lymphoblastoid cell lines

All cell lines used in this study were lymphoblastoid cell lines (LCL’s) with EBV-immortalised B-lymphocytes and they were generated from whole blood samples derived from individuals. Two were obtained as a gift from the Institut Curie, Paris, France, and found to be consistent with a diagnosis of non-Hodgkins lymphoma and Turner’s syndrome (henceforth collectively labelled as NHL-T cell lines; first described by Angéle et al. (36)). Another two cell lines were obtained as gifts from the University of Birmingham and were derived from two distinct AT-patients (henceforth collectively labelled as AT lines). These lines have also been described previously (37).

LCL’s are cells which grow in suspension and do not attach to surfaces within their environment. They were maintained in standard RPMI medium (Sigma) supplemented with 10% Foetal bovine serum (FBS), L-Glutamine and incubated at 37°C with 5% CO_2_. Each cell line was kept at a density of 1x10^5^/ml and split by 1:5 dilutions every 24-48 hrs. Prior to irradiation, 5 ml of cells at a density of 1x10^6^ cells/ml were seeded per flask, per cell line and per dose.

### Isolation of lymphocytes, blood culture and irradiation

This study received ethical approval from the Technological University Dublin Research Ethics Committee (REC number 15-32). Blood was drawn from a total of 23 healthy controls and 19 prostate cancer patients (prior to treatment) as described previously (28, 38). Prostate cancer patients were recruited under a Cancer Trials Ireland trial, CTRIAL-IE (ICORG) 08-17 (NCT00951535). Peripheral blood mononuclear cells were then isolated as described in our previous work (28) through establishment of a blood culture for each blood draw, and isolation of lymphocytes for irradiation through plastic adherence over a 72-hour culture period.

### Sample irradiation

*In-vitro* cultured lymphocytes (for Raman spectroscopy) and blood cultures (for the G2 assay) were both irradiated with an X-ray linear accelerator (LINAC) at SLRON St. Luke’s Hospital, Dublin, as described previously (28). Cells were either sham irradiated or irradiated with a 50 mGy or 500 mGy dose. Samples were then incubated for a 1-hour period at 37 °C before sample preparation for spectroscopy.

### Radiation-induced G2 assay

The G2 assay was performed as described previously (28), using whole blood cultures. Briefly, cells were irradiated with an X-ray LINAC at a dose of 500 mGy, were arrested in metaphase via incubation with colcemid, and were subsequently fixed, lysed and stained using a 3% Giemsa solution. The number of chromatid aberrations was then recorded microscopically for 50 cells per slide. For each donor, a radiation-induced G2 (RIG2) score was recorded as the difference in the number of aberrations seen in the control (0 Gy) sample versus that in the irradiated sample. Examplar images of the chromosomal aberrations observed during this analysis have been published previously (in White at al, and Bryant et al. (37, 39)).

### Raman slide preparation

Following IR exposure, lymphocytes in suspension were first centrifuged at 250 g for 5 minutes. Supernatant was removed and the cells were fixed using 200 µls of 2% paraformaldehyde in phosphate-buffered saline. From the suspension, 40 µl was drop cast onto calcium fluoride (CaF_2_) slides. Paraformaldehyde was removed using disposable graduated pipettes and the slides for Raman spectroscopy were then rinsed in deionised H_2_O for 5 minutes. Washing was performed three times and then the slides were allowed to dry for Raman spectroscopic measurements.

### Raman spectroscopy and post-processing

Raman spectroscopy was performed using a Horiba Jobin Yvon Labram HR800 UV spectrometer. Spectra were acquired with a 660 nm solid-state diode laser delivering 100 mW output power, with a 20 second integration time and averaged across three integrations per spectrum. Spectra were recorded using a diffraction grating ruled with 300 lines/mm giving a spectral resolution of ~2.1 cm^-1^. The confocal hole was set to 150 μm with the grating centered at 1350 cm^-1^. A total of 30 to 50 spectra for each donor, cell line and dose were recorded from the nuclear portion of the cell using a raster scanning methodology described elsewhere (28, 38, 40). The cells were ~8-12 μm in size and each spectrum was recorded from individual cells with a 4x4 μm raster scan of the centre of the cell including both the nucleus and cytoplasm of the cell. All spectra were recorded within two weeks of slide preparation. Spectra were downsampled using intersample averaging to produce approximately 8-15 high SNR spectra per donor, cell line and treatment condition, resulting in a total of 1279 spectra for analysis (28).

All post processing was performed using Matlab version 7.9.0 (R2009b; Mathworks, USA) using the PLS-Toolbox version 6.51 (Eigenvector Research Inc.) and in-house algorithms. Wavenumber calibration, intensity calibration, baseline correction, smoothing and removal of substrate contribution were performed as outlined in our previous studies (28, 38). All spectra were subsequently vector normalized before analysis and were downsampled to provide a reduced dataset, improving the overall signal-to-noise ratio in each spectrum. In total 1279 spectra were used for model development.

### Machine learning

All analysis and machine learning was conducted in Python v.3.10.9 using sci-kit-learn v.1.4.1.

The dimensionality of the spectral data was reduced before the development of classification models using two approaches, PCA and UMAP. PCA is well established in the field, allowing for the reduction of a spectral dataset from a large number of covariant wavenumbers to a, typically, small number of orthogonal basis variables which describe the underlying variance in the data (38, 41–43). While PCA does allow subsequent interpretation of the origin of spectral discrimination between classes, often this approach is insufficient to reduce the data to a manageable component subset (29, 44, 45), with the result that models can be overly complex. Recently it has been demonstrated that advanced manifold approaches, including UMAP (46, 47) can be employed with spectral data for variable reduction (44, 48, 49), which, while not in themselves providing insights into the spectral origin of clustering, are effective in reducing spectral data to a small number of components for cluster visualization and model development.

Classification models were developed using a SVM algorithm with either PCA or UMAP as a dimensionality reduction step. The dimensionality reduction step involved the development of a reduction model on the training set which was then applied to both the training and testing data, in order to prevent model leakage via conditioning.

Hyperparameter optimization was performed using a brute-force grid-search cross-validation approach (with 3 folds) for both the SVM algorithm and the dimensionality reduction approaches as follows:

- SVM hyperparameters: C (regularization parameter) – values 0.1,0.5 or 1; kernel – linear, rbf or sigmoid; gamma (penalty parameter) – scale or auto;
- PCA: number of components varied between 1 and 50;
- UMAP: number of components varied between 1 and 4; minimum distance value – 0.1, 0.5 or 0.7; number of neighbours – 30, 100, 170, 230; distance metric – minkowski, Manhattan, Canberra, cosine, or correlation.

All models were executed for 30 separate classification epochs, with data randomized between training (80% of data) and test sets (20% of data) on each pass. Samples were randomized in such a way to ensure model leakage did not occur and that spectra of cells from a given line or donor did not appear in both the training and test sets. In the case of the cell lines on each pass it was ensured that spectra from one AT line, and from one or other of the NHL-T lines, did appear in the training set with the other appearing in the test set.

## 3 Results and discussion

**Figure 1:**
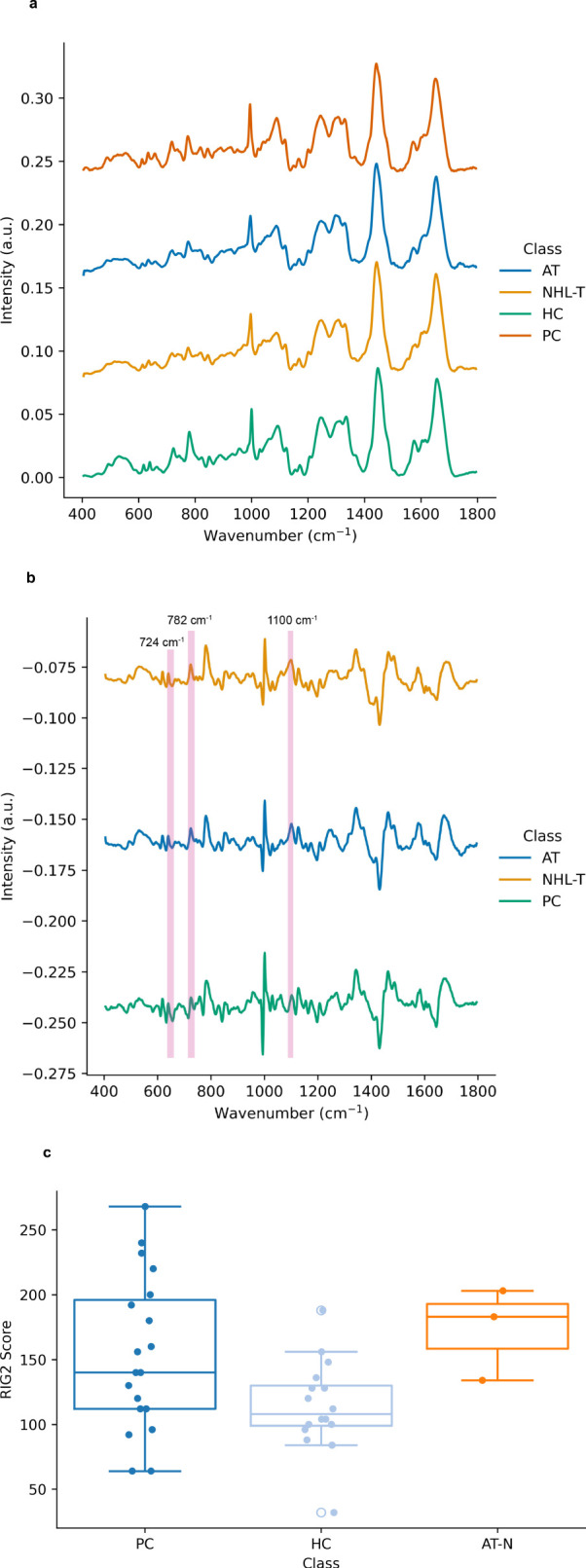
**(A)** Mean Raman spectra of lymphocytes from control samples (0 Gy), including samples drawn from healthy controls (HC), prostate cancer patients (PC), AT-deficient lymphoblastoid cell lines (AT) and two additional immortalized lymphoblastoid cell lines (NHL-T) as described in the main text. Spectra are vertically offset for visual clarity. **(B)** Mean Raman difference spectra for prostate cancer patients (PC), AT-deficient lymphoblastoid cell lines (AT) and reference immortalized lymphoblastoid cell lines (NHL-T) as described in the text. Spectra are offset for visual clarity. **(C)** Distribution of radiation induced G2 scores for each cell subclass. Here the data for the AT and NHL-T cell lines are grouped as ‘AT-N’.

In **Figure 1A** the mean spectra of each of the cell subclasses are depicted. In **Figure 1B** the mean difference spectra of each sample class are calculated, where the mean spectrum of lymphocytes derived from healthy controls are used as a reference. Here there are significant differences in spectral intensity at 724 cm^-1^ (adenine ring breathing mode), 782 cm^-1^ (uracil, cytosine and tyrosine ring breathing modes) and 1100 cm^-1^ (stretching vibrations in -PO_2_^-^), together with a spectral shift of ~3 cm^-1^ between the normal position of the phenylalanine ring breathing mode (~1004 cm^-1^) and that seen in the cancer and radiosensitive cell lines. Taken together these features may have their origins in the differences in DNA repair propensities in each of these subclasses relative to the healthy controls and may therefore be viewed as a spectral biomarker of abnormal repair. As transfection by EBV was used to immortalise these cell lines, this may also contribute to the spectral differences observed in the lymphoblastoid cell lines here.

**Figure 1C** depicts the RIG2 scores measured in these samples (originally partially produced in our previous work (28)), together with RIG2 scores measured for both the healthy control population and the AT and N cell lines (which are grouped together for the purposes of visualisation due to there being RIG2 score data from only 3 cell lines available). While this data exemplifies the range of radiosensitivities which are yielded from cytogenetic approaches in cancer patients, radiosensitive subpopulations and healthy controls, the overlap in the distributions also demonstrates the challenges in using RIG2 scores for the purposes of precision identification of radiosensitive sub-populations.

**Figure 2:**
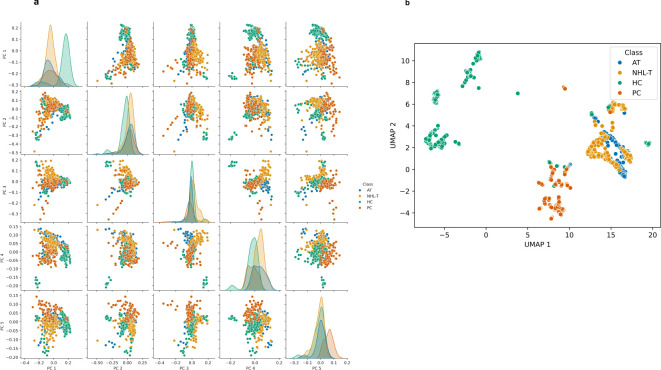
**(A)** Principal component analysis score plot for Raman spectra of the control sample (0 Gy) using a model including the first 5 principal components. This model describes a total of 75% of the spectral variance in the data. **(B)** Score plot depicting the typical distribution of UMAP scores obtained from an embedding model with two components.

**Figure 2A** depicts the PCA scores plot for spectral data at a dose of 0Gy. This PCA model was found to account for 75% of the variance in the spectral data (for reference, a model including the first 10 components was found to describe 86% of the variance in the spectral data). This demonstrates that PCA models account for a substantial proportion of the spectral variance, with a level of clustering seen between scores using many PCs between PC1 and PC5 (**Supplementary Figures S1**, **S2** depict the PCA score plots observed at doses of 50mGy and 500mGy, respectively). However, as the boundary between classes is highly non-linear, this suggests the requirement for deployment of non-linear machine learning approaches for classification here.

**Figure 2B** then depicts a typical plot of UMAP scores obtained from an embedding utilizing two components, again for spectral data observed at a dose of 0Gy. UMAP score plots, unlike PCA score plots, are highly dependent upon the embedding hyperparameters, such that the score plot will differ substantially for other embeddings. As a non-linear embedding approach UMAP has been demonstrated to produce robust embeddings which have been shown to preserve the global structure and continuity of datasets (47, 50). Here it can be seen that this non-linear dimensionality reduction approach can produce embeddings which separate spectral classes such that modelling with a non-linear classification approach is likely to produce higher classification performances.

**Figure 3:**
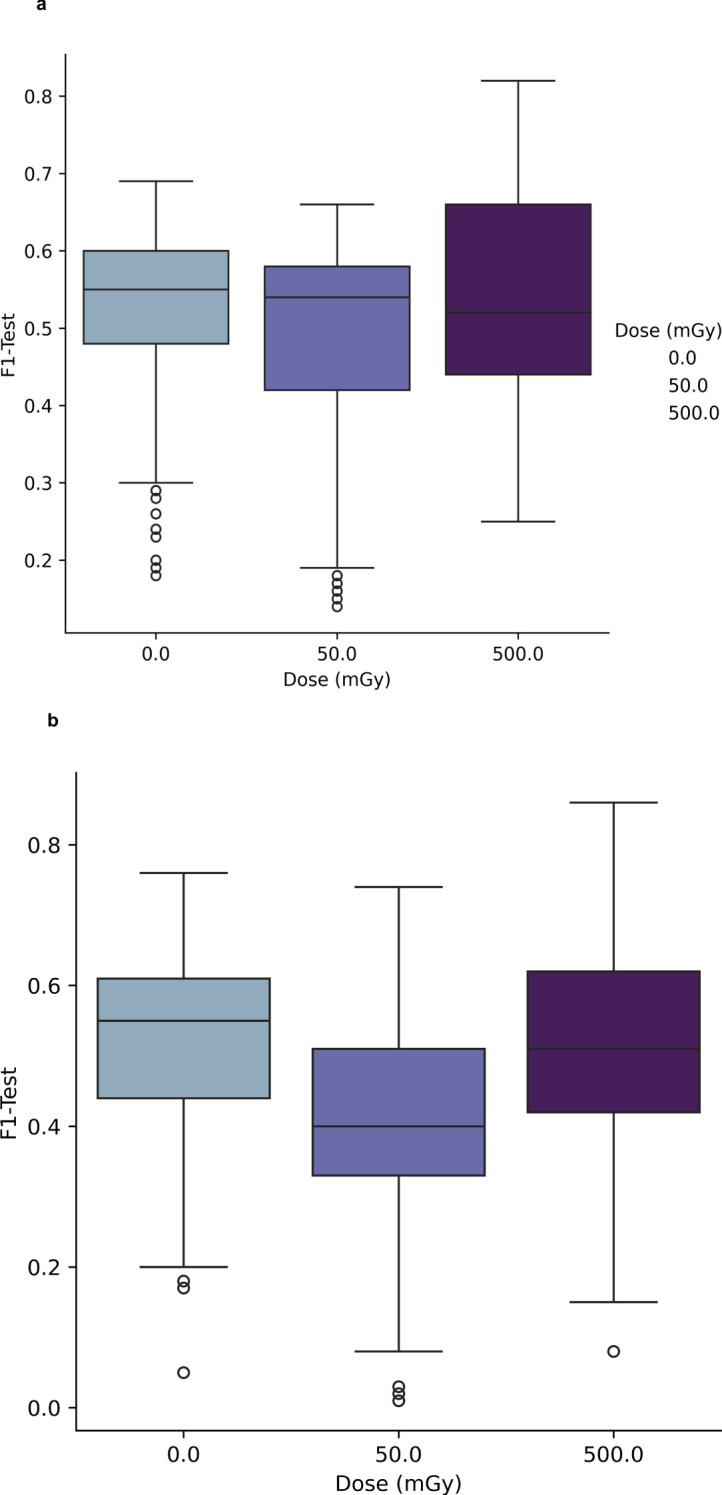
**(A)** Performance of PCA-SVM models as a function of PC and *in-vitro* radiation dose for a 4-class classification model. **(B)** Performance of UMAP-SVM models as a function of *in-vitro* radiation dose for a 4-class classification model.

**Figures 3A** and **B**, the classification performance of the PCA-SVM and UMAP-SVM models are depicted as a function of *in-vitro* radiation dose for a 4-class classification approach (classes – healthy control, prostate cancer, AT-LCL and N-LCL). Modelling performance was expressed as F1-score on the held-out test set, and provided as the macro-average across each class.

For PCA-SVM and UMAP-SVM models, macro F1-score performances were high (μ = 0.96 (σ ± 0.07) and μ = 0.95 (σ ± 0.02), respectively) though these reduced significantly when the tuned models were presented data from the held-out test set, with mean PCA-SVM modelling performance for the test set at 0Gy at F1 = 0.54 (± 0.08) and that for the UMAP-SVM models averaging at F1 = 0.52 (± 0.1) across all hyperparameters. It can be seen from **Figures 3A** and **B** that little variation in modelling performance with either dose or hyperparameter for either model type was observed.

**Figure 4:**
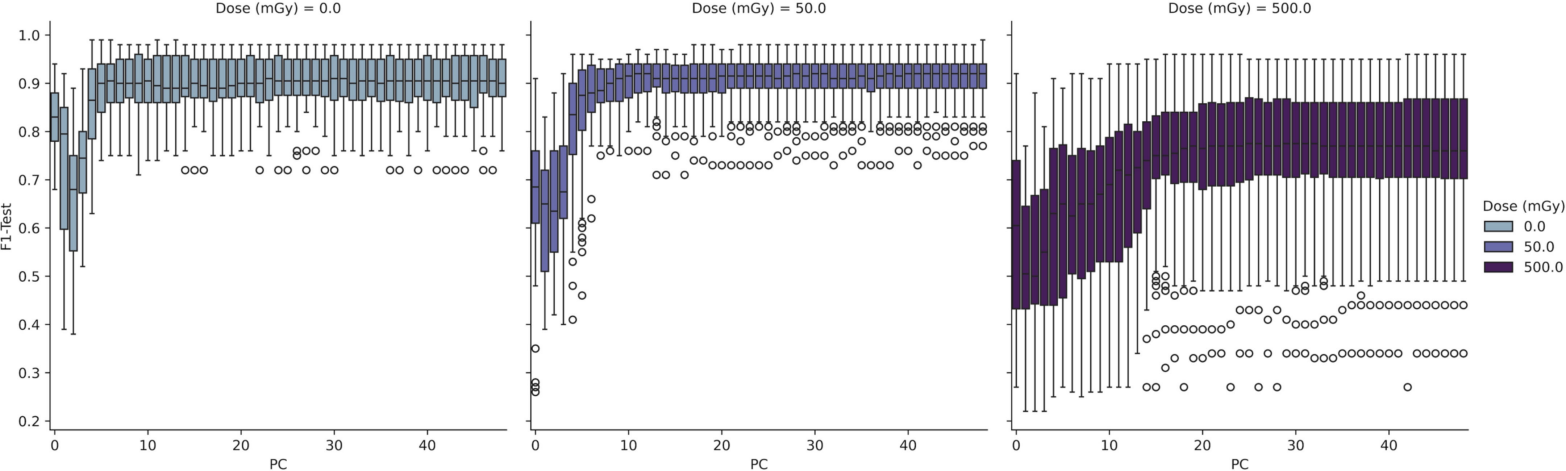
Performance of PCA-SVM models as a function of *in-vitro* radiation dose and model complexity (number of PCs).

**Figure 5:**
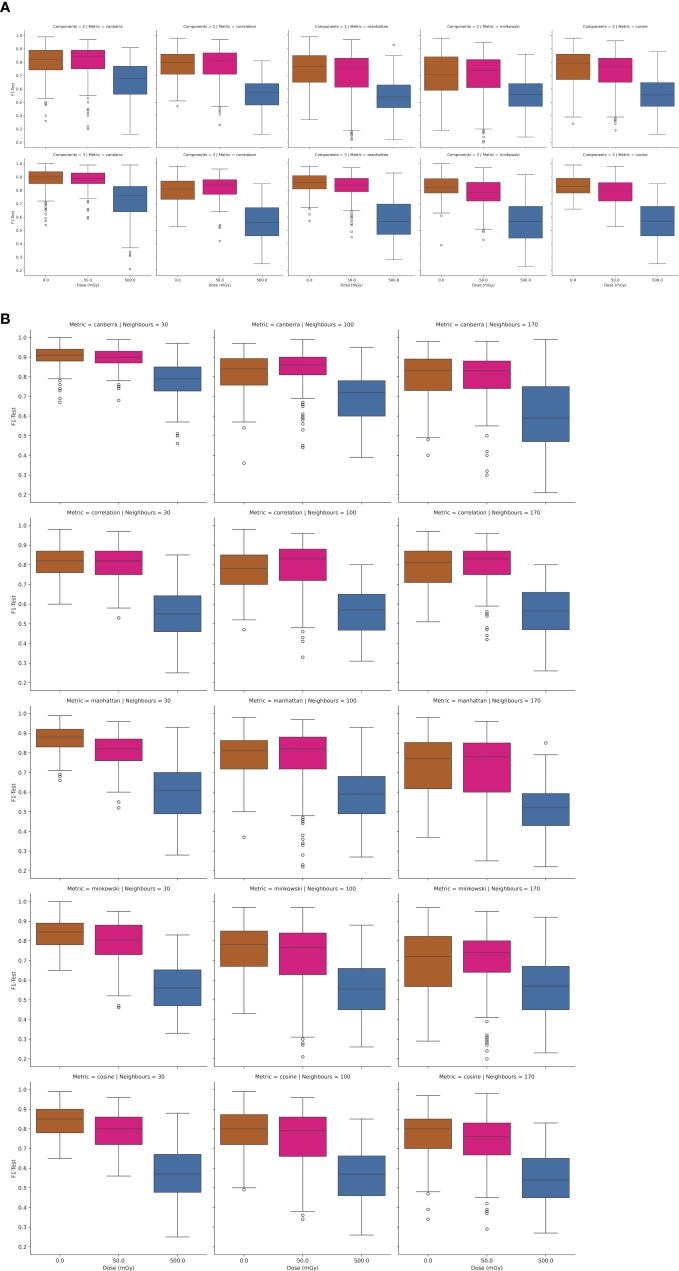
**(A, B)** Performance of UMAP-SVM models as a function of in-vitro radiation dose, UMAP model complexity (number of components, number of neighbours), and distance metric.

To further interrogate the potential to discriminate radiosensitive subpopulations, the AT and N LCL cell lines were grouped together as a unified class and models were then trained separately for discrimination of each class. Results of this analysis are depicted in **Figures 4** and **5**.

In **Figure 4** the test results of a 3-class PCA-SVM classification approach are shown by variation in *in-vitro* radiation dose and number of PCs retained in the model. All models optimize with in the region of 6-7 PCs, with spectral variance increasing with dose, and optimal model performance (mean F1 = 0.89 (σ ± 0.07) observed for models trained on spectra at 0Gy, with model complexity set to 6 PCs.

Likewise in **Figures 5A, B**, the UMAP-SVM model performances are provided with variation in hyperparameter. Modelling performances are again maximized for spectral data at a 0Gy dose, with performance falling with dose. Additionally UMAP-SVM model performances appear to be slightly more robust and parsimonious when developed using the *canberra* metric (with number of neighbours set to 30, components to 3) with a mean prediction performance on the held-out test set of F1 = 0.92 (σ ± 0.06). A two-tailed t-test of the F1-score performances returned by the models at testing revealed that they were statistically significantly different (p<0.025), suggesting that the UMAP embedding of spectral data provides superior classification performance for this use case.

Within the context of this study, which uses liquid biopsies from healthy individuals, those with a confirmed diagnosis of cancer, and those from individuals with a confirmed genetic diagnosis of conditions conferring radiosensitivity, this high classification performance is encouraging.

This result suggests that spectroscopic assays for radiosensitivity may potentially have an important role to play within a pipeline of assays for identification of individuals at risk of adverse effects from exposure to ionizing radiation, either in a clinical or non-clinical context. Within a clinical context, these findings point to the potential of this spectroscopic assay within a pipeline for a-priori segregation of those individuals likely to possess a radiosensitive phenotype, and who therefore require careful monitoring for adverse responses during radiotherapeutic treatment or may be candidates for alternative therapeutic strategies. Importantly, the success of this spectroscopic assay does rely upon careful optimisation of sample preparation for optimal classification performance (34, 43). Equally, establishing coherent, robust linkages between spectral observations and the underlying molecular events which give rise to the spectral phenotype is crucial to provide confidence to biomedical scientists and clinicians for interpretation of the spectroscopic measurement.

While this experimental finding does require validation in a larger cohort, it is potentially important as, despite a significant body of research in this area, a single assay for radiosensitivity has yet to emerge (35). It is therefore likely that any future deployment of this technology in radiation science will involve its use alongside measurements for other characteristics of radiation response.

## 4 Conclusion

In this article, AT lymphoblastoid cell lines established from blood samples taken from two different AT patients, a further one with NHL, and a separate lymphoblastoid cell line from an individual with Turner’s syndrome were used as cellular models of radiosensitivity, together with spectra of lymphocytes drawn from healthy controls and prostate cancer patients. Parallel reference measurements of cellular radiosensitivity were recorded as ground truth metrics, establishing the difficulties in using, for example, cytogenetic metrics such as RIG2 score as an objective measure of radiosensitivity in mixed human populations.

The study has demonstrated the capability of Raman spectroscopy as a tool for the assessment of individual radiosensitivity. Differences in spectral biochemical signatures o cells have been shown to allow for detection and discrimination of cells based on intrinsic factors relating to radiosensitivity. Of course, this work uses only a few biological examples of radiosensitivity as models, and a caveat must be applied to the fact that lymphoblastoid cell lines, rather than lymphocytes drawn from patients, have been used as radiosensitive samples here. Overall however, the study demonstrates clearly the capability of the classification pipeline for the identification of radiosensitive cell subpopulations within a wider heterogenous population.

Using a UMAP-SVM model an F1-score of 0.92 ± 0.06 was observed from classification models using Raman spectra acquired on a 0 Gy (control) sample. Therefore, the incorporation of UMAP spectral decomposition within the classification pipeline, and careful hyperparameter tuning, appears to be a key enabling step providing decomposition of spectral data towards classification by machine learning.

This study has highlighted the potential of a measurement of the Raman spectra of a pre-treatment blood biopsy for identification of radiosensitive subpopulations. However, it is likely that further model training data, together with models which utilize spectral data together with other biological metrics, will be required for high-precision isolation of radiosensitive subpopulations in practice.

## Data Availability

Spectral data acquired from the lymphoblastoid cell lines used in this study are available on Zenodo at the following link: https://doi.org/10.5281/zenodo.14888782. The remaining spectral data may be shared by the corresponding author upon reasonable request.
