# Supplementary material for: Detection of radiosensitive subpopulations ex-vivo with Raman microspectroscopy
Source: Front Oncol. 2025 Feb 27;15:1470431. doi: 10.3389/fonc.2025.1470431 (PMC11903398; doi:10.3389/fonc.2025.1470431)

Figure Captions

Figure S1. PCA score plots for spectral data observed at an *in-vitro* radiation dose of 50mGy.

Figure S2. PCA score plots for spectral data observed at an *in-vitro* radiation dose of 500mGy.

Figure S1

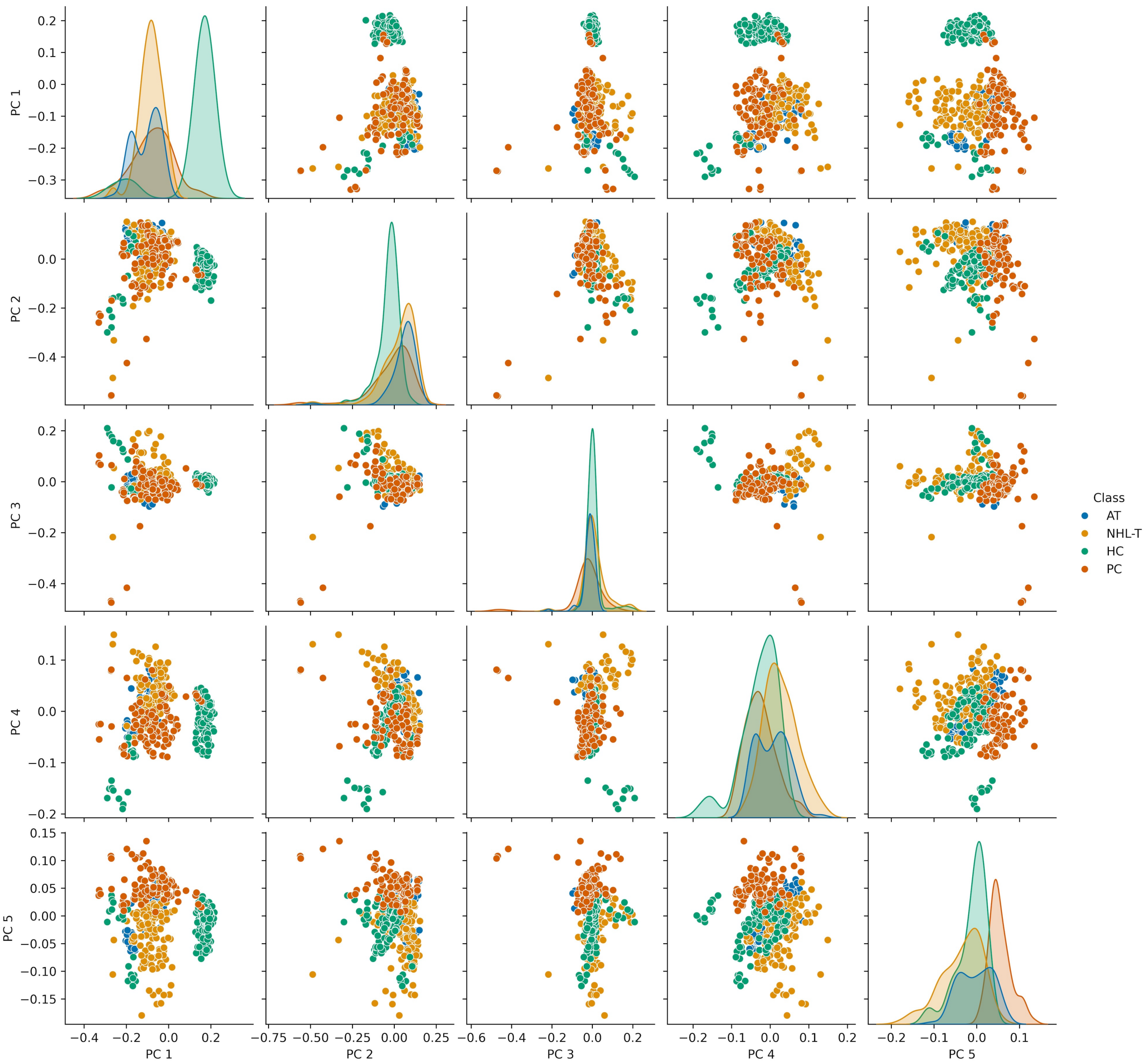

This is a provisional file, not the final typeset article

Figure S2

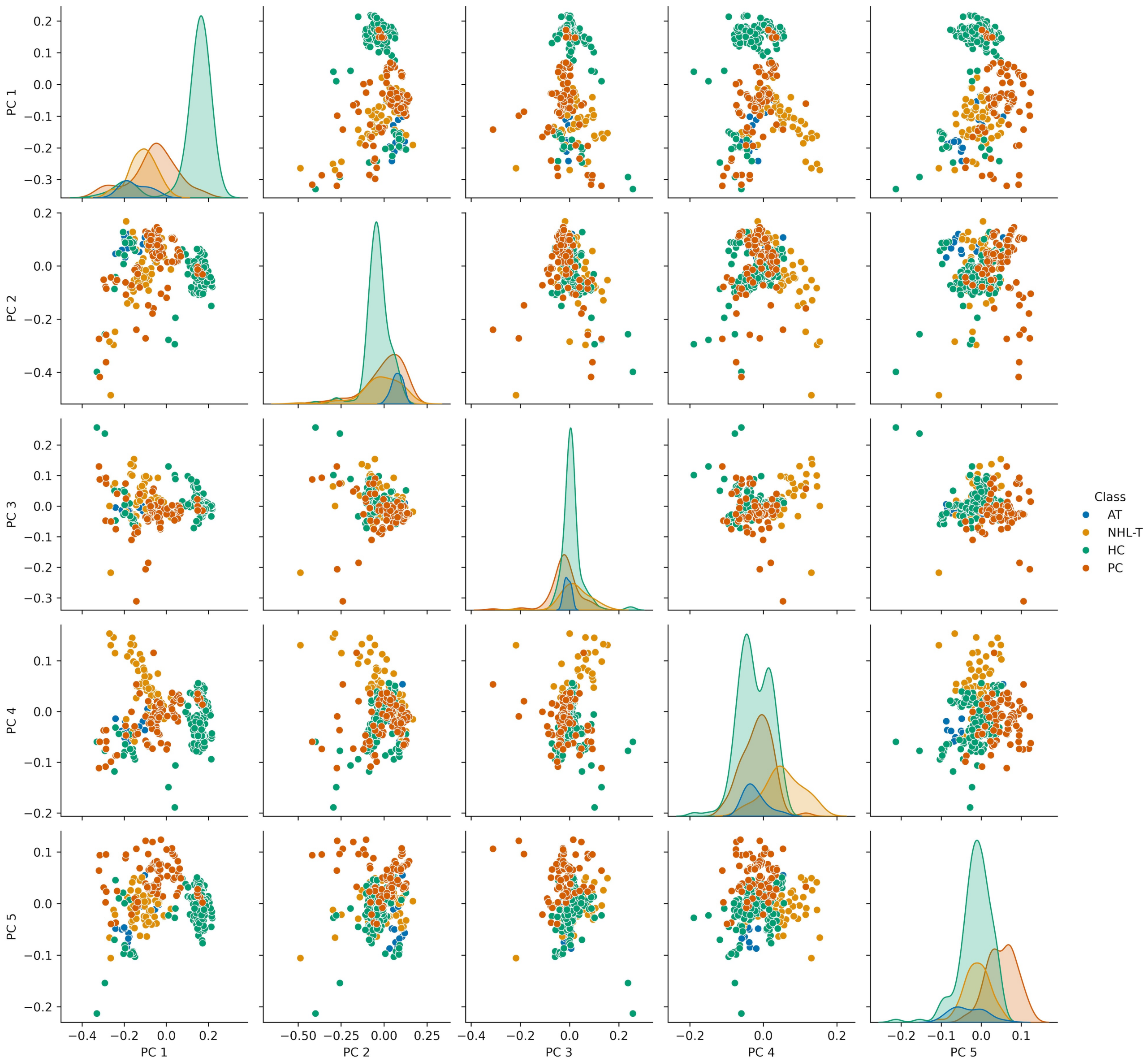

Supplement: Supplementary file 1 [file Image1.pdf]
